# Metal artifact reduction for 3 T MRI-only prostate radiotherapy in patients with hip prostheses: impact on target definition, synthetic CT generation, dose calculation, and fiducial marker identification

**DOI:** 10.1186/s13014-026-02922-w

**Published:** 2026-09-07

**Authors:** Mizgin Coskun, J. Stefan Petersson, Sacha af Wetterstedt, Patrik Brynolfsson, Christian Jamtheim Gustafsson, Adalsteinn Gunnlaugsson, Lars E. Olsson, Carl Siversson

**Affiliations:** 1 Medical Radiation Physics, Department of Translational Medicine, Lund University, Lund, Sweden; 2 Radiation Physics, Department of Hematology, Oncology, and Radiation Physics, Skåne University Hospital, Lund, Sweden; 3 Division of Oncology, Department of Clinical Sciences Lund, Skåne University Hospital, Lund, Sweden; 4 MR Radiation Oncology, GE HealthCare, Helsingborg, Sweden

## Abstract

**Background:**

Magnetic resonance imaging (MRI) offers superior soft-tissue contrast and MRI-only radiotherapy (RT) eliminates uncertainties associated with computed tomography (CT)-to-MRI image registration. An MRI-only RT workflow in patients with hip prostheses remains challenging since delineation, synthetic CT (sCT) generation, and marker identification ideally rely on MR images, which are sensitive to distortions. Metal-artifact reduction sequences (MARS) mitigate metal distortions but often obscure gold markers. The aim of this study is to present a proof-of-concept 3 T MRI-only RT workflow for prostate cancer patients with hip prostheses, using only MARS MAVRIC-SL (GE HealthCare) for delineation, sCT generation and fiducial marker identification.

**Methods:**

Five prostate cancer patients with one or two hip prostheses and intraprostatic fiducial markers underwent CT, standard 3 T MRI and additional MAVRIC-SL MRI. All images were used retrospectively and did not affect patient treatment. Delineation and sCT generation were performed using MAVRIC-SL. Intraprostatic markers were not visible in MAVRIC-SL due to artifact suppression; instead, raw data were reconstructed into spectral-bin images to manually identify the markers. To prevent incoming radiation from passing through the implants and reduce the associated uncertainties in dose calculation and treatment delivery, beam avoidance sectors (AS) were applied. Conventional CT-based avoidance sectors were registered to the sCT and defined as AS1. Prosthesis-induced signal voids in MAVRIC-SL were used to define AS2. Two treatment plans (42.7 Gy, 7 fractions) were created per patient using AS1 and AS2. Plans were compared using dose-volume histogram (DVH) parameters. AS2-plans were recalculated on registered CTs for DVH-comparison and gamma analyses (2%/2 mm and 1%/1 mm with 10% dose threshold) were performed.

**Results:**

Delineation, marker identification and sCT generation were successful using MAVRIC-SL. Median dose differences between AS1 and AS2 remained within ± 0.1% for PTV, while OAR metrics ranged from − 0.4% to + 0.7%. AS2-plans recalculated on CT showed dose differences ≤ 0.9% for PTV and ≤ 1% for OARs. Median gamma pass rates were 99.1% (2%/2 mm) and 96.4% (1%/1 mm).

**Conclusions:**

This study demonstrates the feasibility of an MRI-only radiotherapy workflow at 3 T for prostate cancer patients with hip prostheses, enabling delineation, sCT generation, dose calculation and marker identification from a single MAVRIC-SL acquisition.

**Clinical trial number:**

Not applicable.

**Supplementary Information:**

The online version contains supplementary material available at https://doi.org/10.1186/s13014-026-02922-w.

## Introduction

Prostate cancer is the most common cancer among men in Sweden, accounting for approximately 29% of all male cancer cases, with the peak incidence at ages 75–79 [1]. Radiotherapy (RT) is a standard treatment, administered to about 50% of prostate cancer patients [2]. Magnetic resonance imaging (MRI) provides superior soft-tissue contrast for RT planning. This enables more accurate target delineation and potentially reducing radiation dose to organs at risk (OARs) compared with computed tomography (CT) [3–6]. Treatment planning in conventional radiotherapy workflow relies on CT-MRI registration, a process which introduces geometrical uncertainties [7]. In an MRI-only RT workflow, synthetic CT (sCT) generated from MR image replaces CT image, eliminating image registration uncertainties and all steps, including sCT generation, fiducial marker identification, and delineation, can be performed within the same image geometry [8–12].

In Sweden, a substantial proportion of hip prosthesis procedures are performed in patients aged 75–84 [13], indicating that a considerable share of prostate cancer patients may also have such metallic implants. During treatment planning for patients with metallic hip prostheses, the beams are arranged to avoid going through the metal [14]. In CT images, the geometrical extent of the prosthesis is directly depicted, whereas in MR images, metal appears as a signal void with a volume not matching the exact geometry of the prosthesis [15]. In addition, metal can cause geometrical distortions and signal pile-up in MR images, resulting in artifacts that extend beyond the true prosthesis boundaries [16].

While clinical routines for patients with hip prostheses typically employ either a CT-based or CT-MRI combined workflow, the presence of metal poses a major challenge in an MRI-only RT workflow. Metal-induced artifacts can compromise sCT generation through incorrect assignment of Hounsfield unit (HU) values, thereby affecting dose calculation [17–18]. Despite these challenges, previous studies have demonstrated the feasibility of sCT generation and MRI-only RT workflows in the presence of metallic hip prostheses [19–20]. These have been performed at 1.5 T, for which metal-induced artifacts are less severe compared to 3 T [21]. One study incorporated a metal artifact reduction sequence (MARS), which resulted in a signal void closely matching the actual prosthesis volume [20]. However, metal-induced artifacts from hip resurfacing implants increased from a mean diameter of 77 mm at 1.5 T to 93 mm at 3 T when imaged with a T2-weighted (T2w) turbo spin-echo (SE) sequence [22]. This suggests that MRI-only RT may pose a challenge at 3 T. The performance of MRI-only RT workflows at 3 T in patients with metallic hip prostheses remains largely unexplored, representing an important knowledge gap.

Several techniques have been developed to mitigate metal-induced artifacts, including View Angle Tilting (VAT), Slice Encoding for Metal Artifact Correction (SEMAC) and Multi-Acquisition with Variable-Resonance Image Combination (MAVRIC) [21–23]. A hybrid approach, known as MAVRIC-SL, integrates MAVRIC’s spectral binning with SEMAC’s 3D slice-encoding, thereby reducing both in-plane and through-plane artifacts more efficiently [24].

We hypothesize that an MRI-only RT workflow is feasible for prostate cancer patients with hip prostheses at 3 T. The aim of this study is to present proof-of-concept for such a workflow using a single MAVRIC-SL sequence for delineation of target and OARs, sCT generation, and fiducial marker identification.

## Methods & materials

### Patient group

Ethical approval for this study was obtained from the Regional Ethical Review Board (Dnr 2020–06380). Prostate cancer patients who had intraprostatic fiducial markers, one or two hip prostheses, and who were scheduled for an MRI scan, were recruited. All participants were informed about the study and provided written consent to participate. The study included five prostate cancer patients, of which three had unilateral hip prostheses and two had bilateral hip prostheses. Additionally, five patients without hip prostheses were included for delineation comparison. All patients had intraprostatic fiducial markers inserted as part of their prescribed treatment.

### CT and MRI acquisition

As part of the standard clinical workflow, the patient group with hip prostheses was included in a CT-MRI combined workflow and therefore received both CT and MRI scans prior to radiotherapy. The routine MRI protocol includes a multi-echo gradient echo (MEGRE) sequence and T2w PROPELLER (radial k-space acquisition) sequences acquired in three planes: coronal, sagittal and axial (Figure S1). All MRI examinations were performed with the patient in the radiotherapy treatment position. For the purpose of the study, they also underwent an additional MRI scan with the MAVRIC-SL sequence immediately following the routine MRI during the same scan session. All MRI scans were performed on a 3 T GE Architect scanner (Software SIGNA_LX1.MR30.0_R01_2242.a, GE Healthcare, Milwaukee, WI, USA).

The prescribed CT and MRI images, together with the additional MAVRIC-SL images, were used in the study. All data were handled retrospectively, and the treatment of the patient was not affected. Imaging parameters of the MAVRIC-SL sequence are summarized in Table 1, and additional imaging details are provided in the supplementary section (Figure S2 and Table S1).

**Table 1:** Acquisition parameters for MAVRIC-SL

Parameter	MRI acquisition
Sequence	3D MAVRIC-SL
TR / TE [ms]	4654 / 16
Echo train length (ETL)	48
Bandwidth [Hz/pixel]	694.4
Slice thickness [mm]	2.6
Acquisition matrix	360 × 360
Acquired in-plane resolution [mm]	1.3 × 1.3
Reconstructed in-plane resolution [mm]	0.94 × 0.94
Frequency FOV* [cm]	48
Phase FOV	0.7
Acquisition time [min]	9

^*^FOV= field-of-view

In conventional MRI, spatial encoding relies on the assumption that the Larmor frequency of hydrogen protons varies predictably with applied magnetic field gradients, allowing signal to be assigned to a specific anatomical location. Near metallic implants, local magnetic field inhomogeneities cause protons in surrounding tissue to resonate at frequencies that deviate substantially from the expected Larmor frequency, resulting in signal misregistration and loss that renders adjacent anatomy uninterpretable [16]. MAVRIC-SL addresses this by dividing the frequency spectrum into a series of discrete, narrow-bandwidth acquisitions termed spectral bins [24]. Each individual bin selectively excites and acquires signal from protons resonating within a specific frequency sub-range using a 3D fast spin-echo readout. Because each acquisition spans only a narrow bandwidth, spatial misregistration within that bin is markedly reduced compared to a broadband acquisition. By systematically stepping the bin center frequency across the full off-resonance range introduced by the implant, typically spanning tens of kilohertz, the sequence ensures that tissue at all degrees of off-resonance is captured in at least one bin. The final image is reconstructed by summing the images from all spectral bins on a voxel-by-voxel basis. Tissue that would be lost or displaced in a conventional sequence is thereby recovered, yielding clinically interpretable images of soft tissue immediately adjacent to metallic implants [24]. Before the MAVRIC-SL acquisition, a metal analysis sequence may be performed to determine the required number of frequency-offset bins, with the number of frequency-offset bins directly affecting the subsequent MAVRIC-SL acquisition time. In this study, 19–23 spectral-bin images were acquired with a frequency step of 1 kHz. In the product version of the MAVRIC-SL, the individual spectral bins are discarded and only the combined image is stored.

In order to obtain the individual spectral-bin images, raw data from the MAVRIC-SL acquisitions were reconstructed offline. The offline reconstruction included the standard 3D Cartesian pipeline with parallel imaging, inverse Fourier transform, coil combination, gradient warping correction, and intensity correction (Orchestra, General Electric Healthcare, Milwaukee, WI, USA), which is the same process used on the scanner. However, unlike the standard MAVRIC-SL reconstruction on the MR console, the final bin combination step was omitted in the offline reconstruction. This allowed each bin to be analyzed independently.

### Synthetic CT generation

For every patient, an sCT was generated from MAVRIC-SL using the deep learning-based MRI Planner (v2.7.4, Spectronic Medical, AB, Helsingborg, Sweden). It should be noted that the use of MAVRIC-SL with the current version of MRI Planner was experimental and not within the manufacturer’s approved intended use.

The MRI system geometric distortion in MAVRIC-SL was assessed across the full field of view (FoV) using the commercial GRADE phantom and associated software (Spectronic Medical AB, Helsingborg, Sweden) [25–26]. Ensuring geometric accuracy is critical in an MRI-only RT workflow, since any geometric deviations will be directly propagated to the generated sCT, potentially impacting the resulting treatment delivery.

### Fiducial marker identification

In the standard MRI-only RT workflow for prostate cancer patients at our clinic (without metallic implants), all steps, including fiducial marker identification, sCT generation and treatment planning, are performed using MR images from one single standard T2w acquisition (Figure S1c). However, as the cylindrical fiducial markers (1 mm diameter, 5 mm length) appear as small signal voids, distinguishing them from other voids (e.g., calcifications) can be challenging. Therefore, MEGRE images are used as complementary guidance, as signal voids from fiducial markers increase with echo time [11].

A similar approach was applied in this study. Ideally, the MAVRIC-SL images used for target delineation and sCT generation would allow for direct identification of the fiducial markers. However, since MAVRIC-SL is a metal artifact-reducing sequence, the signal voids caused by the fiducial markers were not reliably visible. Instead, the individual spectral-bin images were used, which contained signal voids because the signal was not filled in through spectral-bin combination, as in the final MAVRIC-SL image. Consequently, intraprostatic markers appeared as small signal voids and could be manually identified in the individual spectral-bin images, using Hero (v 2025.2.0, Hero Imaging, Umeå, Sweden), with MEGRE images providing complementary guidance based on the characteristic appearance of fiducial markers. At the identified positions of the fiducial markers, single high-density voxels were manually inserted in the sCT, to be utilized for patient positioning verification.

### Treatment planning

#### Prostheses delineation

In the standard workflow, the prosthesis is delineated on CT and expanded with a 7 mm margin to account for positioning variations relative to the intraprostatic markers, and patient rotations. The exact physical extent of the prosthesis could not be directly visualized on the MAVRIC-SL images. Instead, the signal void caused by the prosthesis was contoured on the MAVRIC-SL images using a signal intensity range of 0–200, which was empirically chosen based on manual image review.

To assess whether the prosthesis was fully contained within the MR signal void, the CT-defined prosthesis, considered the ground truth, was compared to the signal void on MAVRIC-SL images using a rigid CT-MR registration focused on the prosthesis region.

To validate the findings from the patient data under controlled conditions, phantom measurements were performed. Three boxes, each containing a metallic hip prosthesis embedded in agarose gel [27], were imaged with both MRI and CT (Figure S3). Markers placed outside the boxes and visible in both modalities were used for registration to assess whether the hip prostheses were confined within the signal void.

Based on the phantom measurements and patient observations, an additional margin was applied for the definition of the MR-based avoidance sector. To compensate for the susceptibility-induced artifacts extending beyond the MR signal void the signal void was first expanded isotropically by 5 mm in the distal stem region, inferior to the femoral head. This margin accounted for the largest observed difference between the true implant extent and the MR signal void (see Results, Prostheses Delineation). Subsequently, the expanded signal void was further enlarged by 7 mm, following the same approach used for the CT-based prosthesis contour.

#### Delineation on MAVRIC-SL

Delineation of target was performed on MAVRIC-SL by a radiation oncologist. To assess differences between delineation on MAVRIC-SL images and standard T2w SE images, five patients without hip prostheses were scanned using both sequences. Target delineation was first performed on the MAVRIC-SL images, and after one week, it was repeated on T2w SE images by the same radiation oncologist. The MAVRIC-SL images were rigidly registered to the corresponding T2w images, with the T2w images used as the reference. The resulting transformation was applied to the MAVRIC-SL-derived target contours before comparison with the corresponding T2w target contours using the Dice similarity coefficient (DSC), surface Dice similarity coefficient (sDSC) with a 3 mm tolerance, and the 95th percentile Hausdorff distance (HD95).

#### Dose planning

Treatment plans were created on sCT, in the treatment planning system (TPS) Eclipse (v18.00.00, Varian Medical Systems, Palo Alto, CA, USA) for a target dose of 42.7 Gy in 7 fractions. Plans were generated using a single Volumetric Modulated Arc Therapy (VMAT) full arc with a 1 × 1 mm² dose calculation grid, a photon beam energy of 10 MV, and Anisotropic Analytical Algorithm (AAA, v15.6.05). The look-up table used to convert HU values to relative electron density for dose calculation is provided in Table 2.

**Table 2:** Look-up table provided by the TPS for converting HU values to relative electron density for dose calculation

HU	Relative electron density (relative to water)
-3000	0.000
-1050	0.000
-1000	0.000
100	1.100
1000	1.532
6000	3.920

**Fig. 1:**
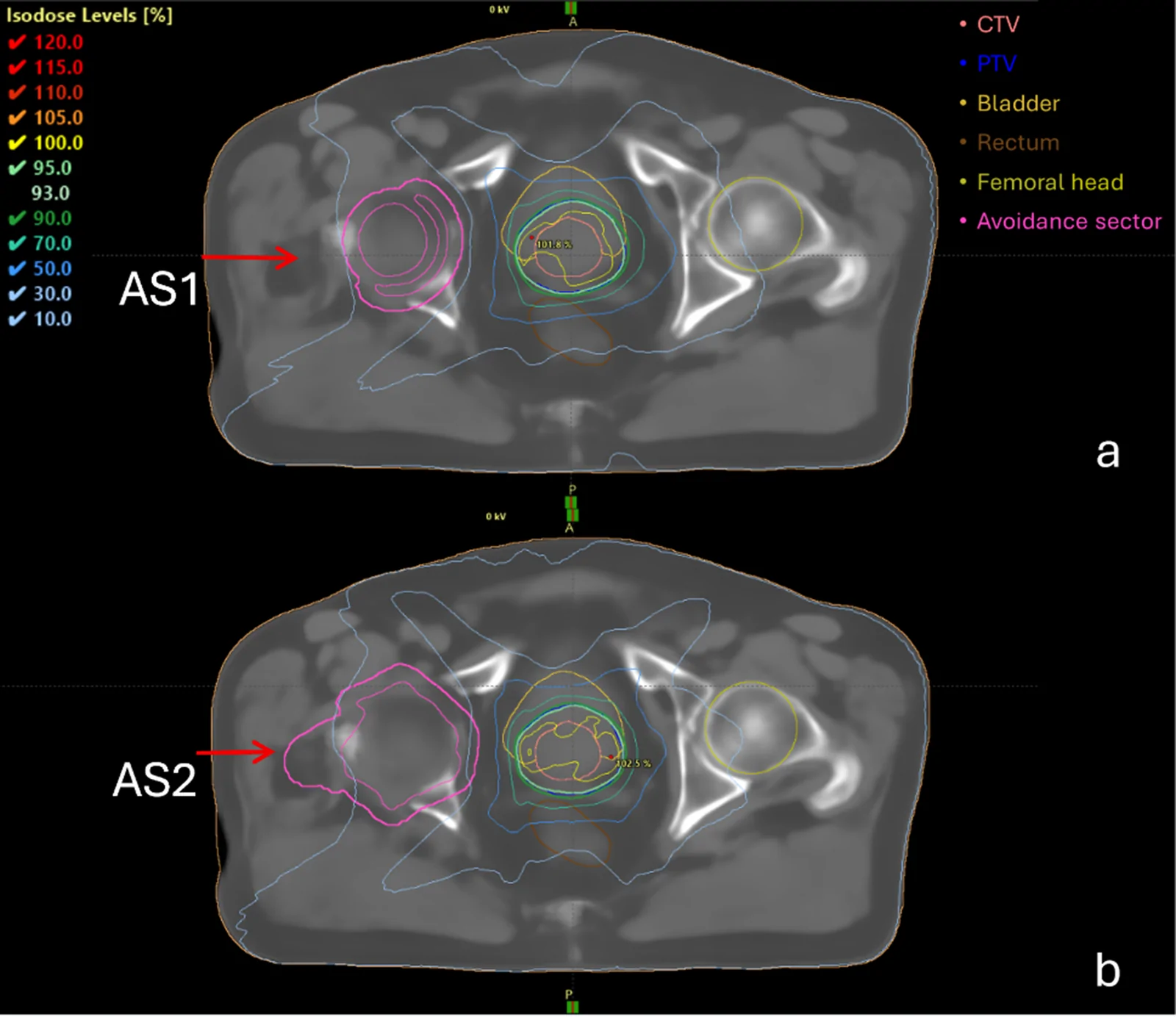
Treatment plans on sCT using different avoidance sectors. (**a**) The hip prosthesis contour expanded by 7 mm was used as an avoidance sector (AS1), as in conventional treatment planning for patients with hip prostheses. (**b**) The signal void expanded first by 5 mm and then by 7 mm, which was used as an avoidance sector (AS2), as the actual metal extent cannot be visualized in an MRI-only workflow, due to signal void. Both treatment plans were optimized using identical target and OARs criteria, with the only difference being the larger avoidance sector in (**b**)

During treatment planning, it is prohibited for the beam to enter through metal; thus, avoidance sectors (AS) have to be applied, during which beam delivery is turned off over a specific range of gantry angles. Two approaches were evaluated: AS1, where the avoidance sector was defined conventionally using the CT-based prosthesis contour expanded uniformly by 7 mm in all directions, and AS2, where it was defined using the MR signal void, which was expanded first by 5 mm and then by an additional uniform 7 mm margin (Fig. 1). For both approaches, the avoidance sector was defined using a beam’s-eye-view (BEV) assessment by identifying the gantry angles at which the radiation beam would pass through the corresponding expanded avoidance structure before reaching the target. These gantry angles were excluded from beam delivery, resulting in patient-specific avoidance sectors. Details of the gantry angles included in each avoidance sector are provided in Table S2.

### Comparison of treatment plans with AS1 and AS2

The two treatment plans, generated on sCT using either AS1 or AS2, were compared using clinical DVH parameters. For the planning target volume (PTV), defined as the clinical target volume (CTV) with a 7 mm margin, the evaluated parameters included the mean dose (D_mean_), near-maximum dose (D_2%_), and near-minimum doses (D_95%_ and D_98%_). In addition, the near-maximum dose (D_2%_) was compared for the OARs, including the rectum, bladder, femoral head, penile bulb, and genitalia.

### Comparison of treatment plans on sCT and CT

**Fig. 2:**
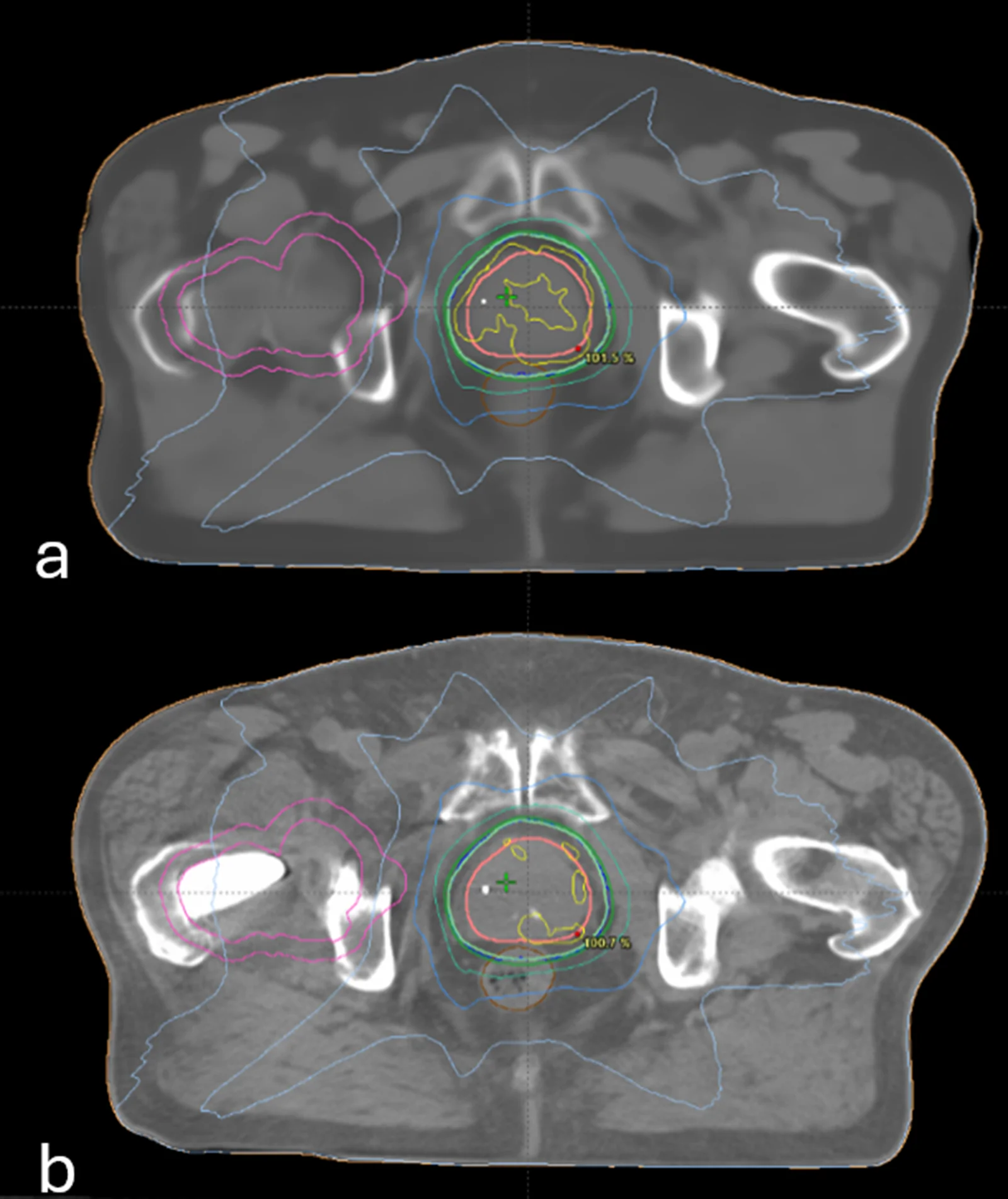
(**a**) Treatment plan optimized on sCT using an MR-derived avoidance sector. (**b**) Corresponding plan recalculated on CT after rigid registration in Hero, using the initial registration from Eclipse

To enable comparison of treatment plans on the sCT and CT, a new rigid registration between the two image datasets was performed in the TPS using automatic bone matching. This ensured consistent alignment of the pelvic anatomy for dose evaluation across all target volumes and organs at risk. The resulting registration parameters were then imported into Hero and used as input for rigid registration and resampling of the CT dataset to the sCT coordinate system. The resampled CT was subsequently imported into the TPS, where the treatment plan optimized on the sCT using AS2 was recalculated on the aligned CT dataset (Fig. 2).

Previously described DVH parameters were compared for the PTV and OARs, between treatment plans calculated on sCT and recalculated on CT. For general comparison of the dose volume, the RT dose plans were exported to Hero for global gamma analyses, with dose-difference (DD) criteria of 1% and 2%, distance-to-agreement (DTA) criteria of 1 mm and 2 mm, a threshold of 10% of the prescribed dose, and the sCT body contour as the analysis mask.

## Results

### Synthetic CT generation

**Fig. 3:**
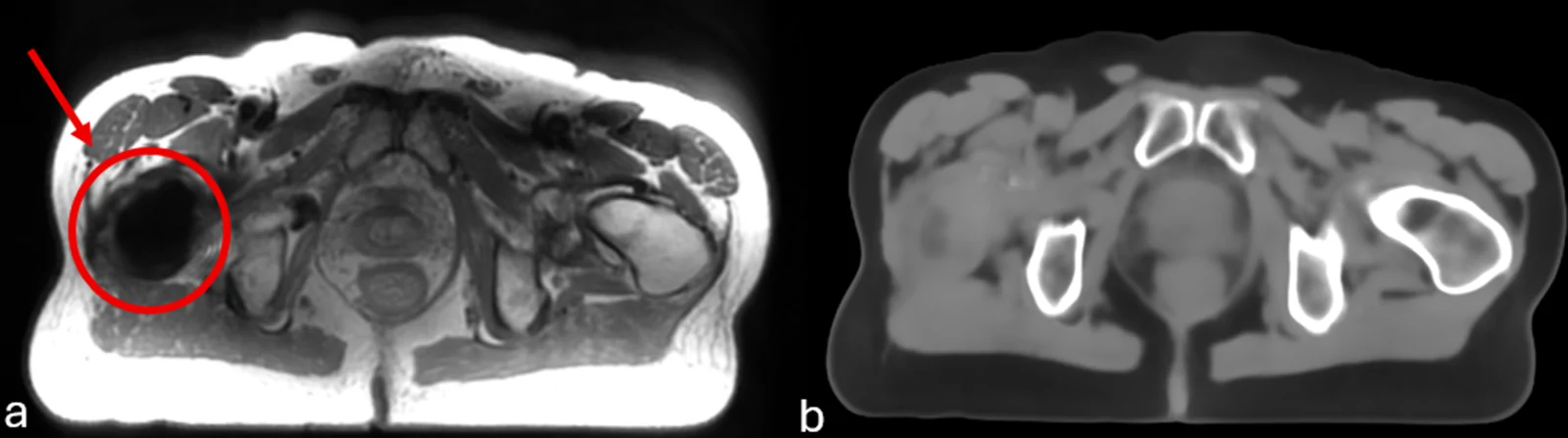
(**a**) MAVRIC-SL image with the red arrow indicating the signal void caused by the hip prosthesis. (**b**) Corresponding sCT image

Synthetic CT images were generated using MAVRIC-SL images (Fig. 3). In the MAVRIC-SL images, the presence of metal implants caused signal voids, which led to incorrect HU assignments within those regions in the sCT. No corrections were applied to the HU values within the signal void regions, since incoming radiation was not allowed to pass through these areas during treatment planning.

Geometric distortions measured using the GRADE phantom remained below 2 mm at all evaluated distances from isocenter (Table 3). Distortions of this magnitude are consistent with geometric accuracy levels previously reported in MRI-guided RT literature [28–29].

**Table 3:** Geometric distortions measured using the GRADE phantom

Geometric distortions [mm]			
Distance to isocenter [mm]	< 100	100–150	150–200
Averaged observed distortion	0.54	0.69	0.91
Max observed distortion	1.06	1.30	1.77

Distortions are reported as average and maximum observed values at different radial distances from the MRI scanner isocenter

### Fiducial marker identification

**Fig. 4:**
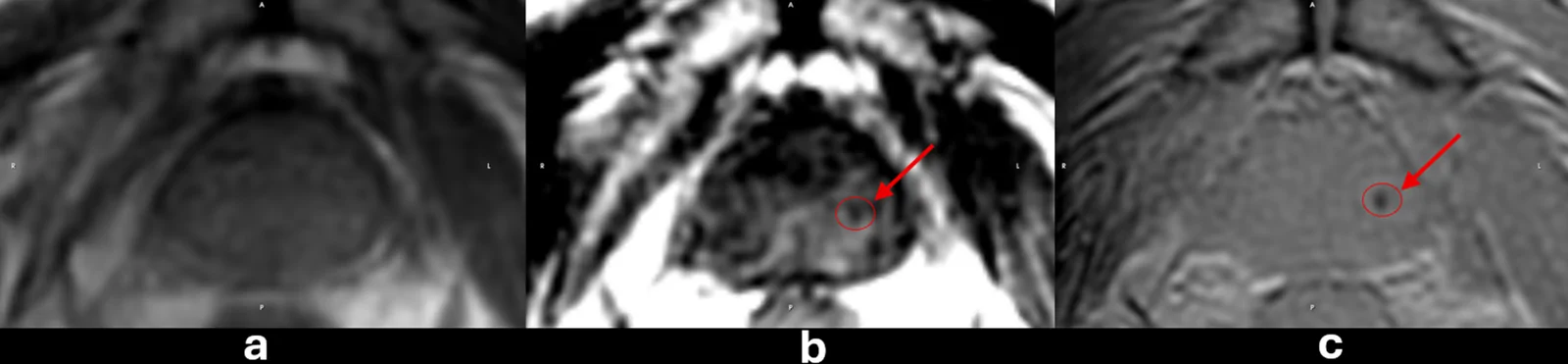
(**a**) MAVRIC-SL image in which the signal void caused by fiducial marker is not visible. (**b**) A single spectral-bin image of MAVRIC-SL, showing the signal void caused by the fiducial marker, indicated by the red arrow and ring. (**c**) Separate MEGRE image at one echo time (TE = 2.4 ms) used to confirm that the signal void indicated by the red arrow and ring is caused by the fiducial marker

The individual spectral-bin images together with MEGRE images as guidance enabled identification of all three markers in every patient (example shown in Fig. 4). The results of the cross-checked distances between the fiducial markers in individual spectral-bin images and MEGRE images are summarized in Table S3. The measured distances were ≤ 2 mm in all cases.

### Treatment planning

#### Prostheses delineation

**Fig. 5:**
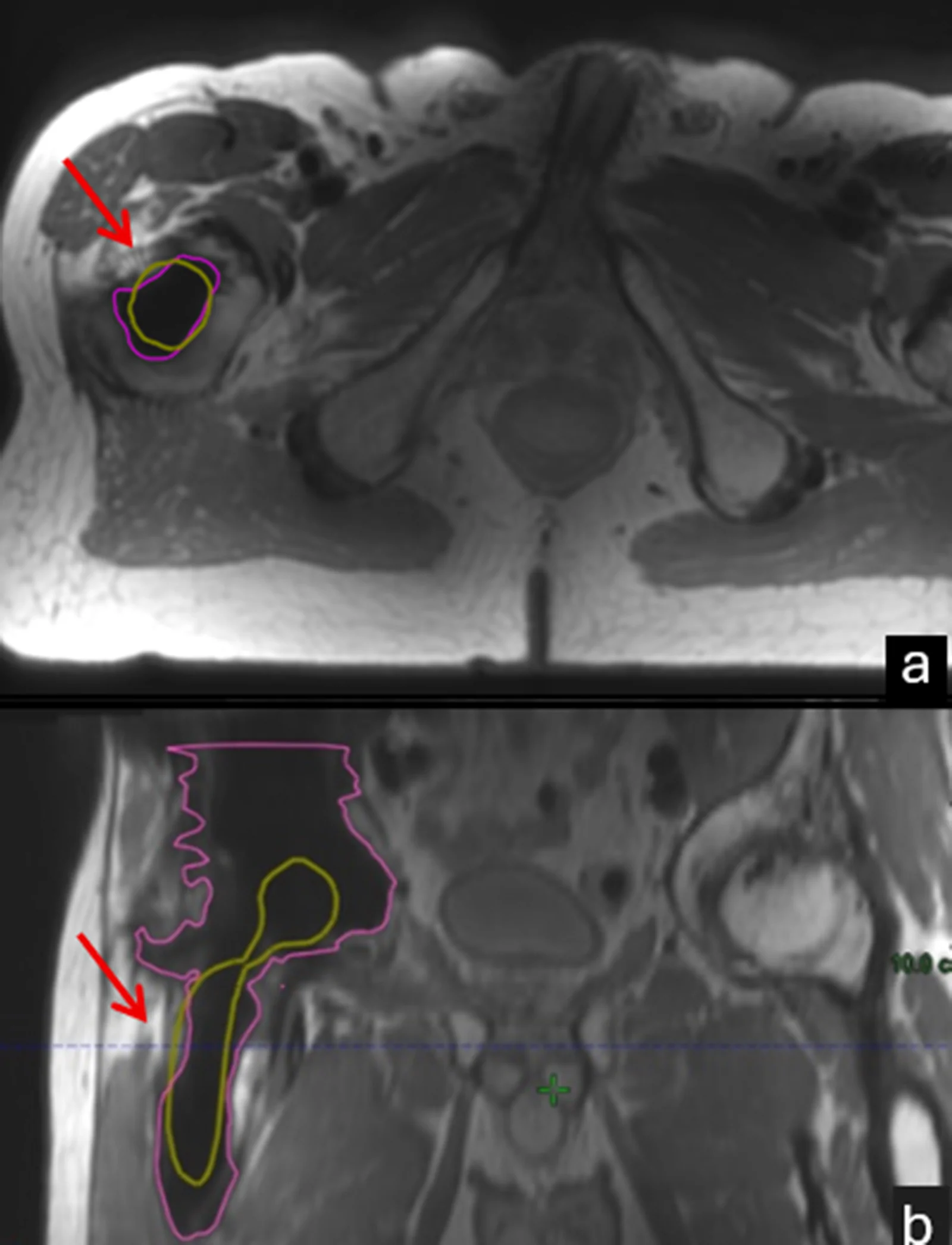
MAVRIC-SL image with the signal void delineated (pink contour). The contour of the hip prosthesis (yellow contour) was delineated on the CT image and transferred to the MR image after a focused prosthesis-region registration. (**a**) Axial plane. (**b**) Coronal plane. Note that the hip prosthesis extends beyond the signal void void along its distal stem, inferior to the femoral head, as indicated by the red arrows

Rigid CT-MR registration restricted to the prosthesis region showed that the metallic implant extended beyond the MR signal void in the distal stem region, inferior to the femoral head (Fig. 5). Across patient datasets, the median extent of the implant beyond the signal void was 4 mm (range 2–5 mm). Notably, regions corresponding to the physical extent of the implant were not consistently represented as signal void on MR but could instead coincide with areas of signal pile-up artifact arising from surrounding tissue. A 5 mm margin was therefore applied to the portion of the implant lying outside the signal void.

The phantom measurements also demonstrated that the metallic implant extended beyond the MR signal void in the stem region, with a median extent of 4 mm (range, 2–5 mm). Notably, the physical extent of the implants was not consistently represented as signal void on MR images and could instead correspond to areas of signal pile-up artifact arising from adjacent tissue (Figure S4).

#### Delineation on MAVRIC-SL

Across the five patient datasets, the median DSC between target delineations on MAVRIC-SL and T2w SE images was 0.89 (range 0.8–0.92) and the median sDSC (3 mm tolerance) was 0.92 (range 0.7–0.98). The lowest sDSC value (0.70) was observed in one patient dataset that showed subtle positional differences between acquisitions. Median HD95 was 2.5 mm (range 1.0–2.8 mm).

### Comparison of treatment plans with AS1 and AS2

Comparison between treatment plans performed on sCT using avoidance sectors AS1 and AS2 showed minor differences in PTV_D95%_ doses, with a median of -0.1% (range − 0.3–0.2%) of the prescribed dose. The treatment plans using AS1 and AS2 met the same clinical criteria. Additional results are provided in Table 4.

**Table 4:** Median signed differences (AS1 − AS2) DVH parameters between treatment plans using two avoidance sectors (AS1 and AS2) for five patients

DVH parameter	Median difference (%)	Range (%) (min – max)
**PTV metrics**		
Mean	+ 0.0	-0.1–0.1
D_98%_	+ 0.1	-0.3–0.2
D_95%_	-0.1	-0.3–0.2
D_2%_	-0.1	-0.4–0.0
**OAR metrics**		
Bladder D_2%_	+ 0.0	-0.2–0.3
Femoral head (left)* D_2%_	-0.3	-1.0–0.0
Penile bulb D_2%_	+ 0.2	0.0–0.4
Genitalia D_2%_	+ 0.7	-0.1–0.8
**Rectum**		
Mean	-0.4	-1.0–0.2
D_2%_	+ 0.0	-0.3–0.5
D_30%_	-0.4	-1.8–0.5
V_70%_	-0.3	-0.7–0.0

*Femoral head structures were present in only three patients

### Comparison of treatment plans on sCT and CT

Comparison between treatment plans based on sCT and recalculated treatment plans on CT showed differences around 1% in PTV_mean_ doses, with a median of 0.8% (range 0.4–1.1%). Additional results are provided in Table 5.

**Table 5:** Median signed differences (sCT − CT) in DVH parameters from treatments plans generated on sCT using AS2 and recalculated on CT for five patients

DVH parameter	Median difference (%)	Range (%) (min – max)
**PTV metrics**		
Mean	+ 0.8	0.4–1.1
D_98%_	+ 0.8	0.4–1.1
D_95%_	+ 0.9	0.4–1.1
D_2%_	+ 0.8	0.3–1.0
**OAR metrics**		
Femoral head (left)* D_2%_	+ 0.1	0.1–0.4
Penile bulb D_2%_	+ 0.2	0.0–0.9
Genitalia D_2%_	+ 0.2	0.1–0.3
Bladder D_2%_	+ 1.0	0.3–1.4
**Rectum**		
Mean	+ 0.4	0.2–0.5
D_2%_	+ 0.7	0.5–1.0
D_30%_	+ 0.6	0.2–0.9
V_70%_	+ 0.5	0.3–0.5

*Femoral head structures were present in only three patients

Gamma analysis yielded gamma pass rates that exceeded 96% for all DD and DTA criteria. Detailed results are provided in Table 6.

**Table 6:** Gamma index pass rates for comparison between treatments plans generated on sCT and treatment plans recalculated on CT

Criteria (DD & DTA)	Median gamma index pass rate (%)	Range (%) (min – max)
2% & 2 mm	99.1	98.4–99.2
1% & 1 mm	96.4	96.3–97.3

## Discussion

In this study, we demonstrated a proof-of-concept for a 3 T MRI-only radiotherapy workflow for prostate cancer patients with unilateral or bilateral metal hip prostheses. Using a single MAVRIC-SL acquisition, we showed the feasibility of target and OAR delineation, sCT generation, while showing that fiducial markers could be visualized in the MAVRIC-SL spectral-bin images.

MAVRIC-SL resulted in metal-induced signal voids that were less severe than those observed on T2w SE images (Figure S5). Despite differences between the imaging sequences, target delineation on MAVRIC-SL showed high agreement with T2w SE contours across all evaluated metrics. The observed agreement was comparable to previously reported values for prostate delineation across imaging modalities and between AI-based and manual approaches [30–32]. The lowest sDSC value, observed in one patient, may partly be explained by patient relaxation causing subtle changes in positioning between acquisitions.

The acquisition time of MAVRIC-SL is longer than that of the standard T2w SE sequence used at our clinic (~ 9 min vs. ~4 min). However, if a metal artifact analysis sequence is performed prior to MAVRIC-SL acquisition, the scan time can be reduced to ~ 7 min. Although MAVRIC-SL is currently not compatible with the AI-based reconstruction method AirReconDL available for the T2w fast spin-echo (FSE) sequence on the GE Architect scanner, the resulting scan time remains comparable to acquisition times prior to the introduction of AI-based reconstruction, as reported by Persson et al. (2020) [33]. Future developments may allow integration of AI reconstruction into MAVRIC-SL, potentially further improving image quality and reducing acquisition time.

MRI Planner is currently optimized to generate sCTs from T2w SE images rather than MAVRIC-SL images. Nevertheless, dose distributions calculated on MAVRIC-SL-derived sCTs showed good agreement with those recalculated on CT. Moreover, the results are consistent with those reported by Koivula et al., who observed comparable gamma pass rates when evaluating sCT-based dose calculations in the presence of hip prostheses on 1.5 T [20]. Furthermore, these results demonstrate that MAVRIC-SL-derived sCTs enable accurate dose calculations for the evaluated prostate radiotherapy plans. This is likely facilitated by the sufficient tissue contrast of the MAVRIC-SL acquisition to identify organ boundaries despite its relatively short echo time of 16 ms. This finding is in accordance with the results of Wyatt et al., who reported accurate dosimetric performance of a commercial sCT algorithm (using MRI Planner at 1.5 T) for the male pelvis, applied to patients with unilateral hip prostheses [8].

Overall, these findings demonstrate the feasibility of MAVRIC-SL for sCT generation for patients with unilateral and bilateral hip prostheses, and the observed minor dose differences are unlikely to be clinically relevant. Previous work by Persson et al. (2017) has shown that such differences are primarily driven by residual registration uncertainties and anatomical differences between MR and CT acquisitions rather than by HU inaccuracies in sCT [12].

The comparison of treatment plans using AS1 and AS2 showed only minor differences in the evaluated DVH parameters. Overall, the larger avoidance sector (AS2) did not substantially affect target coverage or OAR doses, indicating that the increased avoidance sector size did not result in clinically relevant deterioration of overall plan quality. This finding is consistent with Koivula et al., who reported that smaller avoidance sectors resulted in only modest or no improvements in OAR doses [20]. This could be explained by the relatively small difference between the volumes of the AS1 and AS2 contours (Table S2). Determining the appropriate patient-specific avoidance sector may be challenging, as the true implant extent exceeds the boundary of the MR signal void. In this study, we therefore applied the maximum observed difference between the physical extent of metal and the signal void, measured up to 5 mm. Accordingly, the signal void was segmented, and a 5 mm margin was added to the avoidance volume in the distal stem region, inferior to the femoral head. A future direction could be to develop methods for determining patient-specific avoidance sectors needed to avoid the metallic implant during treatment planning.

A limitation of this study is the small cohort of five patients. Nevertheless, the results indicate that an MRI-only prostate radiotherapy workflow in the presence of hip prostheses is feasible. The proposed workflow may also be applicable to other pelvic treatment sites with metallic implants, provided that the MAVRIC-SL images offer sufficient image quality and soft-tissue contrast for target and OARs delineation. If modifications to the MAVRIC-SL acquisition are required to optimize image quality for other pelvic indications, the corresponding sCT generation workflow would require re-evaluation and validation. Moreover, as hip prostheses vary in size and composition, the applicability of the proposed additional 5 mm margin should be evaluated in a larger cohort. Intraprostatic fiducial markers were identified in all patients using offline reconstructed individual spectral-bin images. However, these images contained frequency shifts corresponding to their acquisition offsets, which were not corrected, limiting evaluation of fiducial markers to within the same spectral-bin images. The two most cranial markers could be cross-checked using MEGRE images, whereas the third marker appeared in a separate spectral-bin and could therefore not be validated relative to the other markers using inter-marker distance measurements. For the two cranial markers, distance measurements between spectral-bin and MEGRE images showed only small differences, consistent with previously reported MRI-based fiducial localization uncertainties of approximately 1–2 mm [11].

In this proof-of-concept study, an additional offline reconstruction step from raw data was required for fiducial marker identification. For implementation in clinical routine, a MAVRIC-SL version enabling direct storage of individual spectral-bin images prior to their combination into the final 3D MAVRIC-SL volume at the console would be preferable, although this would require software modification. Such functionality could enable fiducial marker identification in a clinical setting, directly within the product version available on the MR system.

The method used in this study to identify fiducial markers in the MARS MAVRIC-SL sequence is vendor-specific. While similar workflows could potentially be explored on MRI systems from other vendors, challenges in fiducial marker identification are expected with other MARS techniques. In these cases, the approach used in this study would not be applicable, as other MARS sequences do not provide individual spectral-bin images. Alternative strategies would therefore be required.

In addition, although sCT generation from MAVRIC-SL data is technically feasible, it is currently outside the manufacturer’s intended use; therefore, development of an MRI Planner version dedicated to sCT generation using MAVRIC-SL images as input would be beneficial.

## Conclusion

In conclusion, this study supports the feasibility of a proof-of-concept 3 T MRI-only radiotherapy workflow for prostate cancer patients with hip prostheses using a single MAVRIC-SL acquisition. All steps, including target and OAR delineation, avoidance sector definition, sCT generation, dose calculation, and fiducial marker identification, were feasible within a single imaging geometry.

## Supplementary Information

Below is the link to the electronic supplementary material.

Supplementary Material 1

## Data Availability

The datasets generated and/or analyzed during the current study are not publicly available due to ethical/privacy restrictions but are available from the corresponding author on reasonable request.

## Abbreviations

AS: Avoidance sector
BEV: Beam’s-eye-view
CT: Computed tomography
CTV: Clinical target volume
DD: Dose-difference
DSC: Dice similarity coefficient
DTA: Distance-to-agreement
DVH: Dose-volume histogram
HD95: 95th percentile Hausdorff distance
HU: Hounsfield unit
FSE: Fast spin-echo
FoV: Field of view
MARS: Metal-artifact reduction sequences
MAVRIC: Multi-Acquisition with Variable-Resonance Image Combination
MEGRE: Multi-echo gradient echo
MRI: Magnetic resonance imaging
OARs: Organs at risk
PTV: Planning target volume
RT: Radiotherapy
sCT: Synthetic computed tomography
sDSC: Surface dice similarity coefficient
SE: Spin-echo
SEMAC: Slice encoding for metal artifact correction
T2w: T2-weighted
TPS: Treatment planning system
VAT: View angle tilting
VMAT: Volumetric modulated arc therapy
