# Supplementary material for: Metal artifact reduction for 3 T MRI-only prostate radiotherapy in patients with hip prostheses: impact on target definition, synthetic CT generation, dose calculation, and fiducial marker identification
Source: Radiat Oncol. 2026 Sep 7;21:124. doi: 10.1186/s13014-026-02922-w (PMC13551977; doi:10.1186/s13014-026-02922-w)
Supplement: Supplementary file 1 [file 13014_2026_2922_MOESM1_ESM.docx]

# Supplementary

Figure S1: Imaging parameters for the 3 T GE Architect scanner used for the clinical MRI examinations. (a) MEGRE sequence. (b) T2-weighted PROPELLER sequence. (c) Standard T2-weighted sequence.

Figure S2: MAVRIC-SL sequence on 3 T GE Architect scanner. The acquisition time is 9 minutes, which is ~5 minutes longer than the T2 SE sequence currently used in the MRI-only workflow at our clinic for prostate cancer patients without metallic implants. Notably, if metal analysis sequence is performed prior to the MAVRIC-SL acquisition the acquisition time can be reduced to approximately 7 minutes.

Table S1: Parameters for CT image acquisition and reconstruction.

| Scanner model | Siemens Somatom Definition AS+ |
| --- | --- |
| Tube voltage | 120 kV |
| Focal spot (mm) | 1.2 |
| Reconstruction matrix | 512x512 |
| Slice thickness (mm) | 3 |
| In-plane resolution (mm) | 0.98x0.98 |
| Metal artifact reduction algorithm | iterative metal artefact reduction (iMAR) |

Figure S3: Metal prosthesis embedded in agarose gel and placed on a metal grid. The implant consists of titanium and cobalt–chromium. Red arrows indicate the markers used for MR-CT registration.

Table S2: Gantry angles used for the avoidance sectors AS1 (CT-derived) and AS2 (MR-derived). Patients with bilateral hip prostheses had two avoidance sectors, one for each prosthesis.

|  | **Avoidance sectors (degrees)** | |
| --- | --- | --- |
|  | **AS1** | **AS2** |
| **Patient 1** | 22 | 28 |
| **Patient 2** | 18 | 28 |
| **Patient 3** | 22 | 22 |
| **Patient 4** | 24 & 26 | 26 & 28 |
| **Patient 5** | 18 & 16 | 20 & 18 |

Table S3: Distances between the two most cranial intraprostatic fiducial markers measured in the single spectral-bin MAVRIC-SL images and the corresponding MEGRE images for each patient. The difference between the measurements is also shown.

|  | Distance between the two most cranial fiducial markers [mm] | |  |
| --- | --- | --- | --- |
|  | **Individual spectral bin-image** | **MEGRE** | **Absolute difference [mm]** |
| Patient 1 | 23.4 | 22.8 | **0.6** |
| Patient 2 | 29.0 | 28.4 | **0.6** |
| Patient 3 | 29.2 | 31.2 | **2.0** |
| Patient 4 | 23.0 | 22.4 | **0.5** |
| Patient 5 | 26.7 | 28.0 | **1.3** |

Figure S4: Hip prosthesis phantom scanned axially with MAVRIC-SL. The metal implant produces a signal void in the MR image. The green contour represents the hip prosthesis delineated on CT and transferred to the MR image after registration. Note that the prosthesis contour does not fully coincide with the signal void but also overlaps a bright pile-up artifact, indicated by the red arrows. (a) Axial plane. (b) Coronal plane.

Figure S5: (a-b) MAVRIC-SL images from two patients, each with unilateral hip prosthesis. (c) T2w image corresponding to (a). (d) T2w image corresponding to (b). Note the smaller metal-induced signal voids in the MAVRIC-SL images (a-b) compared with the conventional T2w images (c-d).
